# Outcome after endovascular treatment for acute ischemic stroke by underlying etiology: Tertiary experience and meta-analysis

**DOI:** 10.3389/fneur.2023.1065484

**Published:** 2023-04-14

**Authors:** Chunlin Ma, Wenbo Cao, Yang Huang, Qiuyue Tian, Yanfei Chen, Yabing Wang, Jian Chen, Peng Gao, Adam A. Dmytriw, Robert W. Regenhardt, Fei Chen, Qingfeng Ma, Liqun Jiao, Bin Yang

**Affiliations:** ^1^Department of Neurosurgery, Xuanwu Hospital, Capital Medical University, Beijing, China; ^2^Department of Neurology, The First Hospital of Shanxi Medical University, Shanxi, Taiyuan, China; ^3^China International Neuroscience Institute (China-INI), Beijing, China; ^4^Department of Neurology, Yankuang Xinlicheng General Hospital, Jining, Shandong, China; ^5^Beijing Key Laboratory of Clinical Epidemiology, School of Public Health, Capital Medical University, Beijing, China; ^6^Department of Interventional Neuroradiology, Xuanwu Hospital, Capital Medical University, Beijing, China; ^7^Neurointerventional Program, Departments of Medical Imaging and Clinical Neurological Sciences, London Health Sciences Centre, Western University, London, ON, Canada; ^8^Neuroendovascular Program, Massachusetts General Hospital, Harvard Medical School, Boston, MA, United States; ^9^Department of Neurology, Xuanwu Hospital, Capital Medical University, Beijing, China

**Keywords:** mechanical thrombectomy, acute ischemic stroke, endovascular treatment, intracranial atherosclerosis, cardioembolism, different etiology

## Abstract

**Objective:**

To investigate the effect of two major etiologies [intracranial atherosclerotic stenosis (ICAS) and cardioembolism (CE])] on outcomes of acute ischemic stroke (AIS) patients due to large vessel occlusion (LVO) after endovascular thrombectomy (EVT).

**Methods:**

Anterior circulation AIS patients receiving EVT were retrospectively analyzed. Clinical and laboratory data were collected. Clinical outcomes including favorable outcome (90-day modified Rankin Scale 0–2), mortality, intracranial hemorrhage (ICH) and symptomatic ICH (sICH) were compared. A systematic review and meta-analysis was also performed.

**Results:**

A total of 302 AIS patients were included and divided into the ICAS group (86 patients) and the CE group (216 patients). Patients in the ICAS group were younger (62[18.0] vs. 68[19.0] years, *p* < 0.001), more likely to have smoking (52.3% vs. 26.9%, *p* < 0.001) and drinking (52.3% vs. 23.1%, *p* < 0.001) history, and more frequently required rescue therapy (80.2% vs. 4.6%, *p* < 0.001) compared to the CE group. However, favorable outcome (aOR 0.722, 95%CI 0.372–1.402, *p* = 0.336) and mortality (aOR 1.522, 95%CI 0.606–3.831, *p* = 0.371) were not significantly different between the two groups before and after adjustment. The incidence of sICH and ICH were comparable between the two groups before and after adjustment. Systematic review and meta-analysis consisted of 8 eligible studies (7 previous studies and this current study), incorporating 552 ICAS patients and 1,402 CE patients. Favorable outcome was slightly more likely in the ICAS group compared to the CE group (54.2% vs. 46.3%, OR 1.40, 95%CI 1.00–1.96, *I*^2^ = 53.2%). Moreover, the ICAS group had a lower rate of mortality (14.3% vs. 22.2%, OR 0.63, 95%CI 0.46–0.87, *I*^2^ = 0.0%) and ICH (19.5% vs. 31.9%, OR 0.60, 95%CI 0.42–0.84, *I*^2^ = 0.0%) than the CE group, while the two groups were similar in sICH rate (5.9% vs. 6.7%, OR 0.94, 95%CI 0.55–1.60, *I*^2^ = 6.3%).

**Conclusion:**

Etiology was not considered as an important factor in functional outcome, despite the differences in baseline characteristics and technical EVT approach. The current study of anterior circulation AIS-LVO patients supports that outcomes for those with ICAS are not significantly different from those with CE.

## Introduction

Stroke is the second-leading cause of both disability and death worldwide (1). Acute ischemic stroke (AIS) due to large vessel occlusion (LVO) has been one of the primary causes of mortality and morbidity among all stroke patients. Endovascular thrombectomy (EVT) is considered the standard therapy for AIS-LVO in the anterior circulation (2), and its safety and efficacy have been demonstrated in numerous clinical trials (3–8). However, EVT for vessel occlusion caused by intracranial atherosclerotic stenosis (ICAS) is a special concern due to technical difficulty and high requirement of rescue therapy (9).

ICAS is extremely common in Asian countries (1, 10, 11). Although ICAS and cardioembolism (CE) are the two most common etiologies of AIS (11), a difference in outcome of AIS patients due to ICAS and CE after EVT is controversial. One study showed that patients with ICAS were more likely to have favorable outcomes than patients with CE (65% vs. 40.2% *p* = 0.01) (12), but other studies showed patients with ICAS had less favorable outcomes or no significant difference compared to those with CE (12–20). Thus, the aim of this study is to investigate the clinical characteristics of AIS-LVO patients with ICAS and CE and the impact of stroke etiology (ICAS and CE) on outcomes after EVT.

## Methods

### Patient selection

In the present study, we retrospectively reviewed consecutive patients with anterior circulation AIS-LVO who underwent EVT between January 1, 2019 and December 31, 2021 at our tertiary care academic institution. LVO was defined as occlusion of the intracranial internal carotid artery and M1/M2 segment of middle cerebral artery. Patients were included if they met the following inclusion criteria: (1) age ≥ 18 years; (2) AIS-LVO in the anterior circulation; (3) modified Rankin Scale (mRS) score 0–2 before stroke onset; (4) baseline National Institutes of Health Stroke Scale (NIHSS) score ≥ 6; (5) baseline Alberta Stroke Program Early CT score (ASPECTS) 6–10 based on non-contrast CT scan; (6) onset-to-puncture time within 24 h. We excluded patients if they had: (1) incomplete baseline and follow-up data; (2) tandem occlusion or unidentified occlusion; (3) pre-stroke mRS score ≥ 3; (4) baseline NIHSS score < 6; (5) baseline ASPECTS score < 6; (6) not treated with EVT. This study was approved by the institutional review board, and the need for written informed consent was waived based on the study’s retrospective design, de-identified data, and minimal patient risk.

### Identification of ICAS and CE

Angiographic classification was used to distinguish ICAS and CE. ICAS was defined as significant fixed focal stenosis confirmed by last angiography after successful recanalization. Significant stenosis was defined as (1) a degree of fixed stenosis >70%, or (2) a degree of fixed stenosis >50% with perfusion impairment on angiography or evidence of reocclusion tendency after endovascular treatment. ICAS was also defined as successful recanalization of reocclusion after balloon dilation, stenting or medical therapy for intracranial stenosis and vascular endothelial injury/dissection was excluded. CE was defined as no evidence of focal stenosis after thrombolysis or thrombectomy or an achieved full recanalization of the occlusion site confirmed by last intraoperative angiography and short-term repeat CT angiography.

### Clinical and laboratory examination

Clinical, laboratory, imaging and procedural data were extracted. Demographic data included age, sex, hypertension, diabetes mellitus, atrial fibrillation, dyslipidemia, and smoking status. Most patients underwent routine preoperative biochemical examination (neutrophil, lymphocyte, platelet, neutrophil to lymphocyte ratio, platelet to lymphocyte ratio, glucose, triglyceride, total cholesterol, high-density lipoprotein, low-density lipoprotein, etc.) and imaging examination (non-contrast computed tomography, computed tomography angiography and digital subtraction angiography, etc.) during hospitalization. Procedural details were also recorded and collected. Successful recanalization was defined as modified Thrombolysis in Cerebral Infarction (mTICI) ≥2b. All clinical data were evaluated by experienced neurologists.

### Clinical outcomes

All clinical outcomes, including functional outcome, death, intracranial hemorrhage (ICH) and symptomatic ICH (sICH), were collected from a prospective database of patients with acute ischemic stroke. The primary study outcome was favorable outcome at 90 days. Favorable outcome was defined as 90-day mRS of 0 to 2. Secondary outcomes were ICH, sICH and 90-day mortality. The definition of sICH was follow-up imaging evidence of hemorrhagic transformation with NIHSS increase ≥4 within 3 days, according to the European-Australian Cooperative Acute Stroke Study II (ECASS II) (21).

### Systematic review and meta-analysis

This systematic review and meta-analysis was performed, searching four databases (Medline, Science Citation Index Expanded, Embase and the Cochrane Library), to investigate the impact of different etiologies on outcomes after MT. Key terms included acute ischemic stroke, mechanical thrombectomy, endovascular treatment, etiology, intracranial atherosclerosis, cardioembolism, etc. All studies published in English from January 1st, 2015 to June 1st, 2022 were searched. Duplicate studies and irrelevant studies were screened by title and abstract, while relevant studies were evaluated using inclusion and exclusion criteria. Studies in this systematic review included case–control studies, cohort studies and registry studies. All conference abstracts, reviews, observational studies, meta-analyses, and case reports were excluded.

### Statistical analysis

Demographics, clinical, laboratory, imaging, procedural characteristics, and outcomes were compared between the ICAS group and the CE group. The Mann–Whitney U test, χ2 test, and Fisher exact test were used to compare variables. Multivariate logistic regression analyses were used to evaluate the clinical outcomes of functional independence at 90 days, 90-day mortality, ICH, and sICH. Further, clinical outcomes analyses were adjusted for age, sex, CAD, AF, history of smoking, history of drinking, anticoagulation use, NIHSS score, ASPECT score, total cholesterol (TC), low-density lipoprotein (LDL), time of onset to recanalization (OTR), and successful recanalization. All calculations were performed using SAS software, version 9.4 (SAS Institute Inc., Cary, NC, United States). A value of *p* < 0.05 was considered statistically significant.

## Results

Among the 443 patients primarily screened in the intervention group, 141 were excluded. The 302 patients included in the present study were divided into the ICAS group (86 patients) and the CE group (216 patients). The details of demographic and baseline characteristics of the patients are shown in Tables 1, 2, respectively.

**Table 1:** Baseline characteristics of patients.

	Total, *N* = 302	ICAS, *N* = 86	CE, *N* = 216	*p*
Demographics				
Age, median (IQR)	65.0 (19.0)	62 (18.0)	68.0 (19.0)	<0.001^*^
Female, *n* (%)	115 (38.1)	16 (18.6)	99 (45.8)	<0.001
HTN, *n* (%)	199 (65.9)	62 (72.1)	137 (63.4)	0.152
DM, *n* (%)	80 (26.5)	26 (30.2)	54 (25.0)	0.352
Dyslipidemia, *n* (%)	64 (21.2)	25 (29.1)	39 (18.1)	0.035^*^
CAD, *n* (%)	73 (24.2)	7 (8.1)	66 (30.6)	<0.001^*^
AF, *n* (%)	94 (31.1)	8 (9.3)	86 (39.8)	<0.001^*^
Smoking, *n* (%)	103 (34.1)	45 (52.3)	58 (26.9)	<0.001
Drinking, *n* (%)	95 (31.5)	45 (52.3)	50 (23.1)	<0.001^*^
Stroke history, *n* (%)	55 (18.2)	12 (14.0)	43 (19.9)	0.226
Antiplatelet use, *n* (%)	58 (19.2)	12 (14.0)	46 (21.3)	0.144
Anticoagulation use, *n* (%)	30 (9.9)	3 (3.5)	27 (12.5)	0.018^*^
Clinical characteristics				
Intravenous thrombolysis, *n* (%)	115 (38.1)	28 (32.6)	87 (40.3)	0.212
NIHSS, median (IQR)	15 (7)	13 (7)	16 (7)	0.002^*^
ASPECT score, median (IQR)	9 (2)	9 (2)	8 (2)	0.047^*^
Occlusion site, *n* (%)				0.123
ICA	103 (34.1)	28 (32.6)	75 (34.7)	
MCA-M1	157 (52.0)	51 (59.3)	106 (49.1)	
MCA-M2	42 (13.9)	7 (8.1)	35 (16.2)	
Right side, *n* (%)	127 (42.1)	37 (43.0)	90 (41.7)	0.829
Good collaterals, *n* (%)	103 (34.1)	30 (34.9)	73 (33.8)	0.857

HTN, hypertension; DM, diabetes mellitus; AF, atrial fibrillation; CAD, coronary artery disease; NIHSS, National Institutes of Health Stroke Scale; ASPECT, Alberta Stroke Program Early CT score; ICA, internal carotid artery; MCA, middle cerebral artery; TOAST, the Trial of Org 10,172 in Acute Stroke Treatment classification; LAA, large artery atherosclerosis; IQR, interquartile rang. *p < 0.05.

**Table 2:** Comparison of laboratory test and procedural information between two etiologies.

	Total, *N* = 302	ICAS, *N* = 86	CE, *N* = 216	*p*
Laboratory test				
Neutrophil, 10^9^/L, median (IQR)	6.6 (3.9)	6.6 (4.0)	6.6 (4.1)	0.510
Lymphocyte, 10^9^/L, median (IQR)	1.2 (0.9)	1.2 (0.7)	1.2 (1.0)	0.771
Platelet, 10^9^/L, median (IQR)	212 (77)	221 (86)	206 (41)	0.124
NLR, median (IQR)	5.5 (6.3)	5.8 (5.6)	5.4 (6.8)	0.577
PLR, median (IQR)	163 (123)	167 (109)	162 (131)	0.631
Glucose, mmol/L, median (IQR)	7.2 (3.4)	7.5 (3.3)	7.1 (3.4)	0.735
TG, mmol/L, median (IQR)	1.1 (1.0)	1.2 (1.0)	1.1 (1.0)	0.360
TC, mmol/L, median (IQR)	4.3 (1.4)	4.5 (1.1)	4.2 (1.4)	0.030*
HDL, mmol/L, median (IQR)	1.2 (0.4)	1.1 (0.4)	1.2 (0.4)	0.239
LDL, mmol/L, median (IQR)	2.6 (1.2)	2.8 (0.9)	2.6 (1.2)	0.029*
Apo-A, g/L, median (IQR)	1.2 (0.3)	1.2 (0.3)	1.2 (0.3)	0.584
Apo-B, g/L, median (IQR)	0.9 (0.3)	0.9 (0.3)	0.8 (0.3)	0.014*
Procedure				
General anesthesia, *n* (%)	62 (20.5)	19 (22.1)	43 (19.9)	0.671
First-line choice				0.735
ADAPT	154 (51.0)	43 (50.0)	110 (51.4)	
Combined	116 (38.4)	32 (37.2)	82 (38.9)	
SR	32 (10.6)	11 (12.8)	21 (9.7)	
Rescue therapy	79 (26.2)	69 (80.2)	10 (4.6)	<0.001*
Balloon dilation alone		17 (24.6)	—	
Stenting ± balloon dilation		40 (58.0)	—	
Intra-arterial medications		23 (33.4)	10 (4.6)	
OTD, min, median (IQR)	252 (211)	268 (1274)	240 (187)	0.058
DTP, min, median (IQR)	125 (47)	128 (59)	125 (56)	0.147
PTR, min, median (IQR)	38 (28)	38 (25)	36 (25)	0.496
OTR, min, median (IQR)	405 (206)	468 (318)	393 (182)	0.015*
Successful recanalization (mTICI 2b-3)	276 (91.4)	80 (93.0)	196 (90.7)	0.523

NLR, neutrophil to lymphocyte ratio; PLR, platelet to lymphocyte ratio; TG, triglyceride; TC, total cholesterol; HDL, high-density lipoprotein; LDL, low-density lipoprotein; Apo, apolipoprotein; OTD, time from onset to door; PTR, time from puncture to recanalization; OTR, time of onset to recanalization; mTICI, the modified Thrombolysis in Cerebral Infarction score. *p < 0.05.

Among the 86 patients in the ICAS group, 16 were female (18.6%) and the mean age was 62 (±18.0) years. Smoking (52.3% vs. 26.9%, *p* < 0.001) and drinking (52.3% vs. 23.1%, *p* < 0.001) was more frequent in the ICAS group, whereas atrial fibrillation (9.3% vs. 39.8%, *p* < 0.001) and coronary artery disease (8.1% vs. 30.6%, *p* < 0.001) were more frequent in the CE group. No significant differences existed in biochemical data between the ICAS and CE groups, except TC (4.5 vs. 4.2 mmol/l, *p* = 0.03), LDL (2.8 vs. 2.6 mmol/l, *p* = 0.029), and Apo-B (0.9 vs. 0.8 g/l, *p* = 0.014). Successful recanalization was achieved in 276 patients (91.4%), and no difference existed between the two groups (93.0% vs. 90.7%, *p* = 0.523). However, rescue therapy was required of 69 patients in the ICAS group (17 were Balloon dilation alone, 40 were Stenting ± balloon dilation, 23 were Intra-arterial medications). 10 patients in the CE group routinely administered intra-arterial medications (tirofiban) by the experienced neurosurgeon because of higher risk of thrombosis after surgery. However, it does not affect the identification of the etiology of AIS.

Univariable analyses and multivariable analysis of functional outcome is shown in Table 3. In the total cohort, 276 patients (91.4%) had successful recanalization, and 145 patients (48.0%) had functional independence at 90 days. The mortality rate of patients was 16.6%. The rate of ICH and sICH was 27.8 and 10.3%, respectively. Successful recanalization was analyzed before and after adjustment (by age, female, CAD, AF, history of smoking, history of drinking, anticoagulation use, NIHSS score, ASPECT score, TC, LDL, OTR, rescue therapy); there was no significant difference observed in the ICAS group vs. the CE group (93.0% vs. 90.7%, OR 1.361, 95%CI 0.527–3.509, *p* = 0.525; aOR 0.540, 95%CI 0.137–2.128, *p* = 0.378). Regarding functional outcomes, the ICAS group had similar proportion of favorable outcome (54.7% vs. 45.4%, OR 1.451, 95%CI 0.879–2.398, *p* = 0.146) and a trend toward lower sICH risk (4.7% vs. 12.5%, OR 0.341, 95%CI 0.116–1.007, *p* = 0.052) compared to the CE group. There were no significant differences in favorable outcome (aOR 0.722, 95%CI 0.372–1.402, *p* = 0.336) and sICH (aOR 0.714, 95%CI 0.212–2.404, *p* = 0.586) after adjustment for confounding factors including age, female, CAD, AF, history of smoking, history of drinking, anticoagulation use, NIHSS score, ASPECT score, TC, LDL, OTR, and successful recanalization. In addition, the rates of mortality and ICH were also not significantly different comparing ICAS and CE before and after adjustment.

**Table 3:** Univariable analysis and multivariable analysis of functional outcomes.

	Total (*n* = 302)	ICAS (*n* = 86)	CE (*n* = 216)	OR	95%CI	*p*	aOR	95%CI	*p*
Favorable outcome, *n* (%)	145 (48.0)	47 (54.7)	98 (45.4)	1.451	0.879–2.398	0.146	0.722^α^	0.372–1.402	0.336
Mortality, *n* (%)	50 (16.6)	10 (11.6)	40 (18.5)	0.579	0.275–1.218	0.150	1.522^α^	0.606–3.831	0.371
ICH, *n* (%)	84 (27.8)	21 (24.4)	64 (29.2)	0.785	0.442–1.391	0.407	1.140^α^	0.592–2.193	0.695
sICH, *n* (%)	31 (10.3)	4 (4.7)	27 (12.5)	0.341	0.116–1.007	0.052	0.714^α^	0.212–2.404	0.586
Successful recanalization	276 (91.4)	80 (93.0)	196 (90.7)	1.361	0.527–3.509	0.525	0.540^β^	0.137–2.128	0.378

^α^Adjusted by age, female, CAD, AF, history of smoking, history of drinking, anticoagulation use, NIHSS score, ASPECT score, TC, LDL, OTR, successful recanalization. ^β^Adjusted by age, female, CAD, AF, history of smoking, history of drinking, anticoagulation use, NIHSS score, ASPECT score, TC, LDL, OTR, rescue therapy. ICH, intracranial hemorrhage; sICH, symptomatic intracranial hemorrhage.

**Figure 1:**
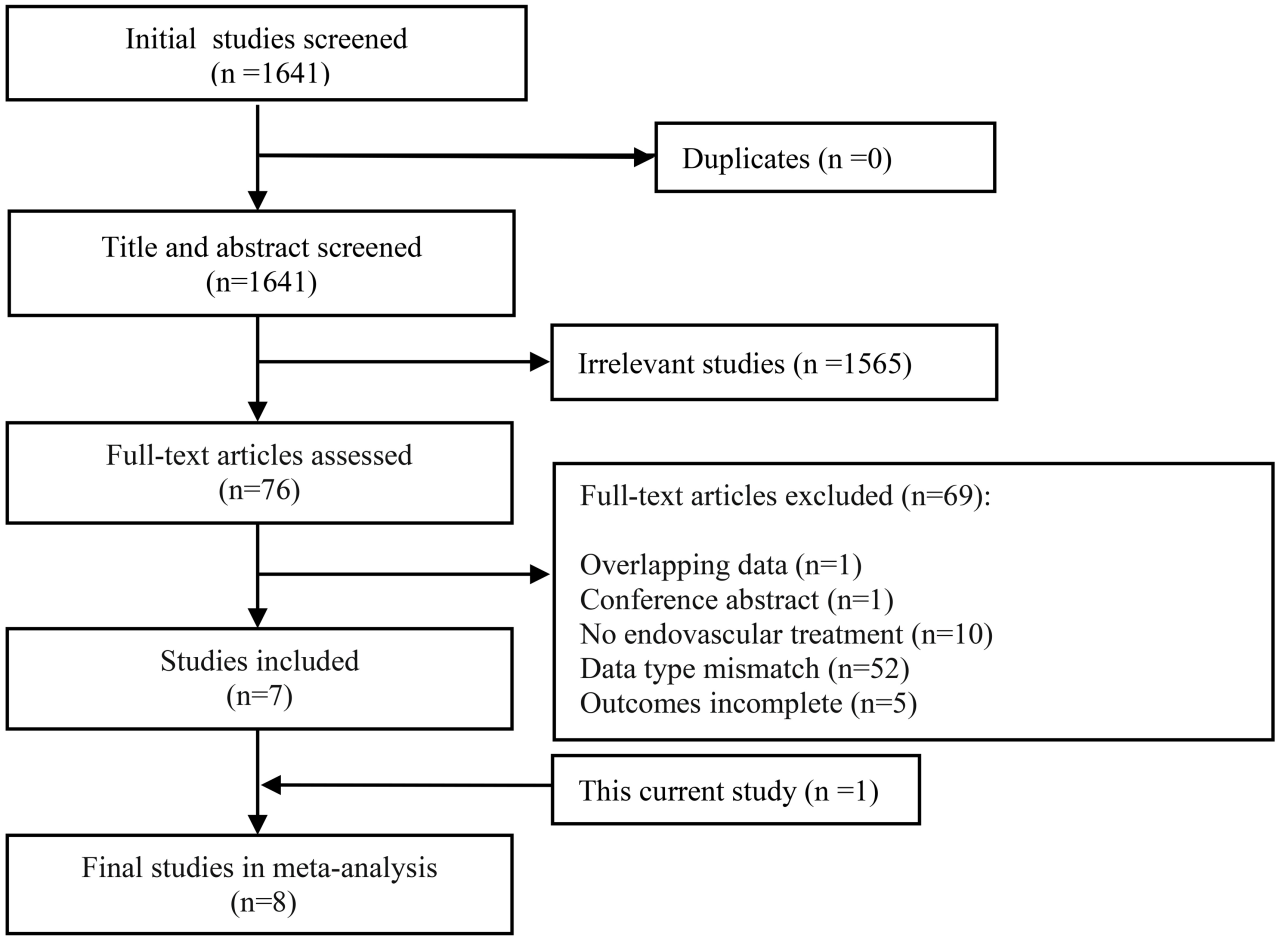
Flow diagram of literature selection for meta-analysis.

In our meta-analysis, the initial search yielded 1,641 studies. A total of 7 eligible previous studies (15, 18, 20, 22–25) were identified meeting the inclusion and exclusion criteria. Of note, studies with vague definitions of other non-ICAS etiologies were eliminated. Our single center data was also included in the meta-analysis. The flow of study selection and details of included studies are shown in Figure 1 and Table 4, respectively.

**Table 4:** Baseline characteristics of patients in meta-analysis.

	2018 Imahori et al.		2018 Jia et al.		2018 Lee et al.		2019 Deng et al.		2019 Guglielmi et al.		2021 Kim et al.		2021 Yang et al.		2022 Ma et al.	
Publication time	2018		2018		2018		2019		2019		2021		2021		2022	
Country	Japan		China		Korea		China		Netherlands		Korea		China		China	
Demographic characteristics																
	**ICAS (*n* = 39)**	**CE (*n* = 11)**	**ICAS (*n* = 47)**	**CE (*n* = 93)**	**ICAS (*n* = 99)**	**CE (*n* = 421)**	**ICAS (*n* = 35)**	**CE (*n* = 46)**	**ICAS (*n* = 190)**	**CE (*n* = 476)**	**ICAS (*n* = 39)**	**CE (*n* = 66)**	**ICAS (*n* = 45)**	**CE (*n* = 45)**	**ICAS (*n* = 86)**	**CE (*n* = 216)**
Age (years) Mean ± SD,Median (IQR)	70 (64–78)	79 (74–85)	60 (52–67)	66 (58–74)	64.8 ± 13.7	68.2 ± 12.4	56.69 ± 11.41	66.30 ± 10.14	69 (62–77)	76 (66–83)	70 (62–79)	71.5 (62.5–79)	62.18 ± 9.41	61.36 ± 12.47	62 (18.0)	68.0 (19.0)
Male, *n* (%)			35 (74.5)	51 (54.8)	60 (60.6)	208 (49.4)	27 (77.1)	21 (45.7)	127 (67)	223 (47)	24 (61.5)	38 (57.6)	38 (84.4%)	31 (68.9%)	70 (81.4)	117 (54.2)
Vascular risk factors																
Hypertension, *n* (%)	7 (65)	25 (64)	29 (61.7)	50 (53.8)	59 (59.6)	257 (61.0)	21 (60.0)	23 (50.0)	83 (44)	290 (61)	19 (48.7)	37 (56.1)	29 (64.4%)	20 (44.4%)	62 (72.1)	137 (63.4)
Diabetes mellitus, *n* (%)	4 (36)	6 (15)	6 (12.8)	8 (8.6)	27 (27.3)	96 (22.8)	5 (14.3)	3 (6.5)	27 (15)	89 (19)	12 (30.8)	26 (39.4)	11 (24.4%)	5 (11.1%)	26 (30.2)	54 (25.0)
Smoking, *n* (%)			22 (46.8)	38 (40.9)	31 (31.3)	84 (20.0)	17 (48.6)	15 (32.6)			14 (35.9)	20 (30.3)	23 (51.1%)	18 (40%)	45 (52.3)	58 (26.9)
Drinking, *n* (%)													7 (15.6%)	5 (11.1%)	45 (52.3)	50 (23.1)
Dyslipidemia, *n* (%)	1 (9)	6 (15)	2 (4.3)	5 (5.4)			1 (2.9)	2 (4.3)			3 (7.7)	8 (12.1)	3 (6.7%)	2 (4.4%)	25 (29.1)	39 (18.1)
Atrial fibrillation, *n* (%)	2 (18)	2 (18)	12 (25.5)	46 (49.5)	24 (24.2)	267 (63.4)					2 (5.1)	39 (59.1)	5 (11.1%)	25 (55.6%)	8 (9.3)	86 (39.8)
CAD, *n* (%)					7 (7.1)	53 (12.6)									7 (8.1)	66 (30.6)
Baseline ASPECTS score, mean ± SD, median (IQR)	10 (9–10)	8 (7–10)	9 (8–10)	9 (9–10)	8 (5–9)	7 (4–9)	10 (8–11)	9 (9–10)	8 (7–10)	9 (7–10)			9 (9–10)	10 (8–10)	9 (2)	8 (2)
Baseline NIHSS score,Mean ± SD,median (IQR)	8 (7–20)	17 (10–23)	14 (12–18)	17 (14–21)	15 (11–19)	17 (13–21)	14 (12–17)	17 (13–22)	16 (12–19)	16 (12–20)	8 (5–12)	13 (7.75–17)	12.0 (8.0–16.0)	12.0 (7.5–15.0)	13 (7)	16 (7)
Occlusion site																
ICA, *n* (%)			15 (31.9)	28 (30.1)	21 (21.2)	170 (40.4)	9 (25.7)	13 (28.3)	93 (49)	87 (19)	9 (23.1)	23 (34.8)			28 (32.6)	75 (34.7)
MCA, *n* (%)											30 (76.9)	43 (65.2)				
MCA M1, *n* (%)			28 (59.6)	47 (50.5)	75 (75.8)	205 (48.7)	23 (65.7)	25 (54.3)	89 (47)	290 (64)					51 (59.3)	106 (49.1)
MCA M2, *n* (%)			4 (8.5)	18 (19.4)	3 (3.0)	46 (10.9)	3 (8.6)	8 (17.4)	8 (4)	68 (1)					7 (8.1)	35 (16.2)
IV tPA	3 (27)	5 (13)	6 (12.8)	18 (19.4)	46 (46.5)	235 (55.8)	6 (17.1)	10 (21.7)	166 (87)	291 (61)	15 (38.5)	35 (53)	17 (37.8%)	12 (26.7%)	28 (32.6)	87 (40.3)
Process time					68 (49–97)	55 (40–83)			73 (50–102)	60 (40–90)						
Time from onset to door (min)			148 (75–260)	123 (68–188)			145 (72–221)	96 (49–150)			199 (108–426)	116 (61–199.25)			268 (1274)	240 (187)
Time from door to puncture (min)			113 (70–166)	109 (60–159)			120 (65–173)	90 (58–159)							128 (59)	125 (56)
Time from onset to puncture (min)	320 (117–489)	137 (72–255)			320 (245–560)	235 (165–374)			207 (165–270)	210 (160–270)			302.0 (196.5–538.5)	301.0 (195.5–442.0)		
Time from puncture to recanalization (min)			68 (42–112)	51 (35–81)			65 (42–88)	44 (34–66)			42 (34–71)	69 (46.75–88)	63.0 (32.0–81.5)	39.0 (28.0–75.5)	38 (25)	36 (25)
Time from onset to recanalization (min)			365 (265–480)	284 (226–406)			365 (263–472)	255 (193–318)							468 (318)	393 (182)
General anesthesia			19 (40.4)	24 (25.8)			21 (60.0)	36 (78.3)					36 (80%)	36 (80%)	19 (22.1)	43 (19.9)
Successful recanalization (mTICI ≥ 2b)	7 (64)	38 (94)	45 (95.7)	90 (96.8)	76 (76.8)	335 (79.6)	34 (97.1)	45 (97.8)	96 (52)	261 (56)	35 (89.7)	60 (90.9)	44 (97.8%)	41 (91.1%)	80 (93.0)	196 (90.7)

CAD, coronary artery disease; NIHSS, National Institutes of Health Stroke Scale; ASPECT, Alberta Stroke Program Early CT score; ICA, internal carotid artery; MCA, middle cerebral artery; MCA-M1, M1 section of MCA; MCA-M2, M2 section of MCA; mTICI, the modified Thrombolysis in Cerebral Infarction score; IQR, interquartile rang; SD, Standard deviation.

**Figure 2:**
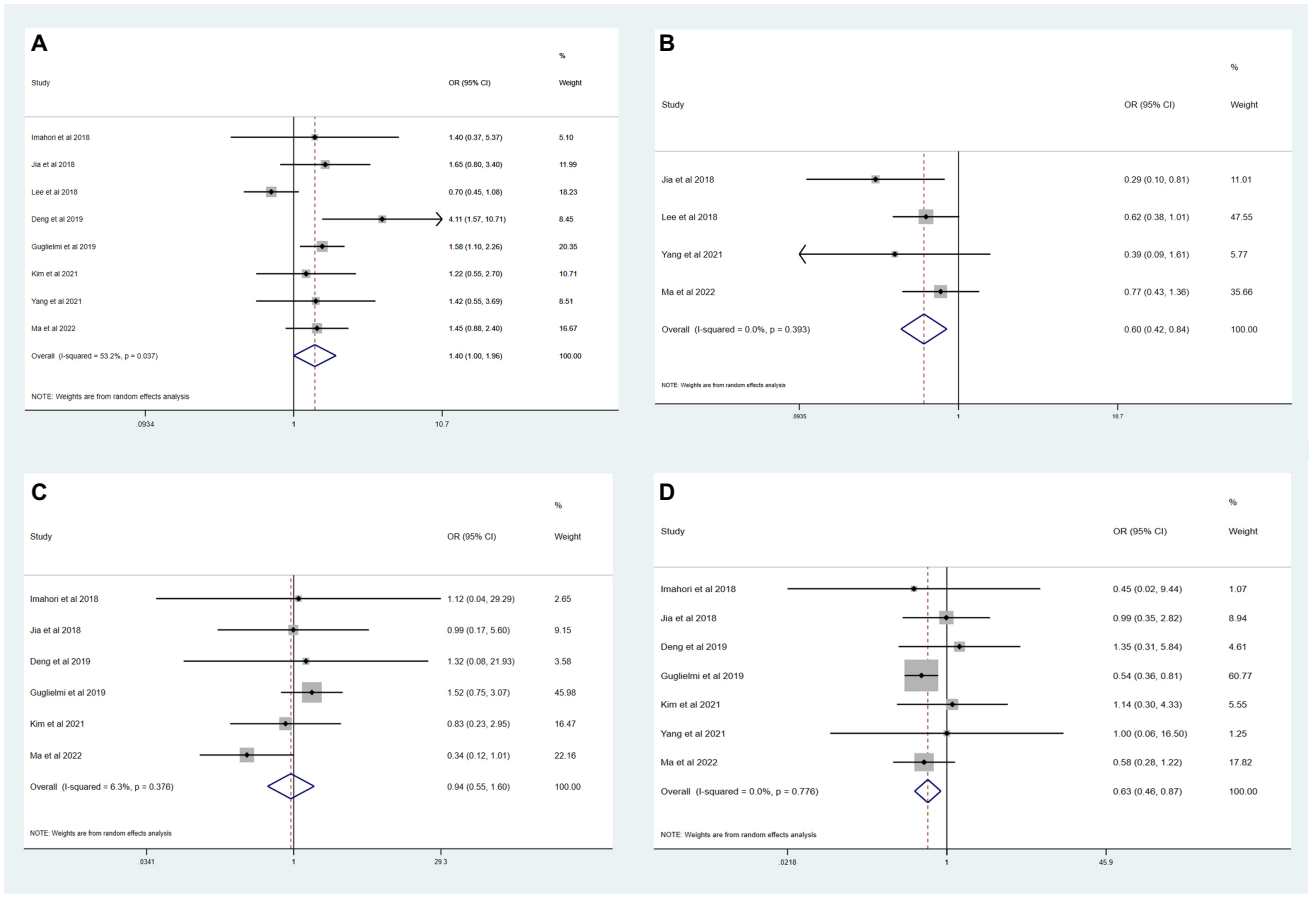
Meta-analysis of etiology impact on thrombectomy outcomes. **(A)** Favorable functional outcome. **(B)** ICH. **(C)** sICH. **(D)** Mortality.

**Figure 3:**
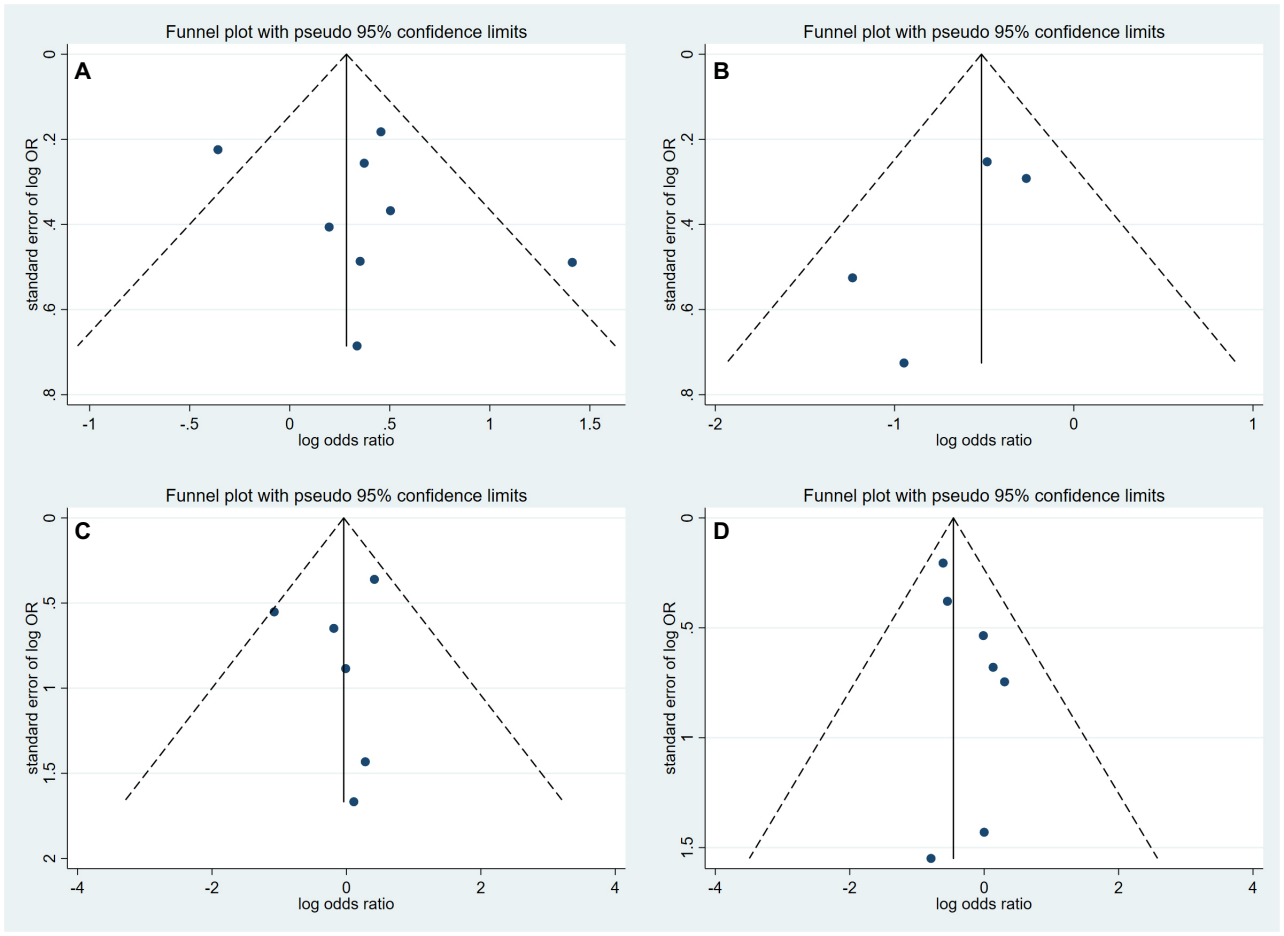
Funnel plot of meta-analysis of thrombectomy outcomes. **(A)** Favorable functional outcome. **(B)** ICH. **(C)** sICH. **(D)** Mortality.

The results of the meta-analysis of the impact of different etiologies on EVT outcomes are shown in Figure 2. The funnel plot of the meta-analysis is shown in Figure 3. The rate of favorable outcome was slightly higher in the ICAS group compared to the CE group (54.2% vs. 46.3%, OR 1.40, 95%CI 1.00–1.96, *I*^2^ = 53.2%). Meanwhile, the ICAS group had a lower rate of ICH (19.5% vs. 31.9%, OR 0.60, 95%CI 0.42–0.84, *I*^2^ = 0.0%) and mortality (14.3% vs. 22.2%, OR 0.63, 95%CI 0.46–0.87, *I*^2^ = 0.0%) compared to the CE group. There was no significant difference in the sICH rate comparing groups (5.9% vs. 6.7%, OR 0.94, 95%CI 0.55–1.60, *I*^2^ = 6.3%).

## Discussion

In this study, we investigated the effect of ICAS vs. CE etiology on outcomes after EVT among anterior circulation AIS-LVO patients. There were some differences in demographics comparing ICAS and CE patients, including age, sex, atrial fibrillation, and smoking history. However, even with more frequent rescue therapy and longer time from onset-to-recanalization, patients with ICAS showed comparable rates of favorable functional outcome and mortality compared with those with CE.

The treatment of ICAS-related LVO by EVT is complex and controversial, as immediate re-occlusion often occurs after initial recanalization potentially due to platelet aggregation at the site of the ICAS occlusion. This directly results in an increase in the use of rescue therapy and in the procedural time (13, 26). However, ICAS patients may achieve comparable successful recanalization rates to CE patients, even though ICAS is associated with a higher likelihood of rescue therapy (14, 15, 17, 20, 27). Therefore, prognosis and other outcomes may be affected by different etiologies of AIS.

In our study, some demographic differences existed between patients with ICAS and CE. Patients in the CE group were older and more likely to have atrial fibrillation history than those in the ICAS group, which supports the hypothesis that atrial fibrillation, more prevalent among elderly patients, is the most common mechanism of thrombogenesis (28). This phenomenon has also been observed in previous studies (18, 23, 25, 29). ICAS patients, in the present study, tended to be males and were more likely to have less healthy lifestyles, such as smoking and drinking, and to have comorbidities, such as coronary artery disease. These tendencies have also been observed in previous studies (14, 17, 27). However, other studies have shown no difference in age and sex comparing the two etiologies (13, 14, 17). Additionally, the ICAS group had a lower average NIHSS score and a higher average ASPECTS score than the CE group. This might be explained by an attenuation of clinical severity by pre-established collateral due to *in-situ* stenosis (ICAS 71% vs. CE 60%, aOR 1.67, 95%CI 1.17–2.39) (22).

Several studies have previously compared the effects of different etiologies on outcomes after EVT, but the data are limited overall, and there are conflicting results. Two studies showed that ICAS patients tended to have lower rates of successful recanalization and less favorable outcomes (13, 16). However, the majority have shown that favorable outcomes and mortality were not significantly different between ICAS and CE etiologies, findings that corroborate our study. In the accompanying systematic review and meta-analysis, we found that ICAS patients were more likely to have favorable outcomes with less mortality and ICH. In contrast, Yoon et al. (12) showed that the ICAS group had more successful recanalization and favorable outcomes compared to the CE group, possibly related to improved collateral circulation due to chronic stenosis in the target vessel (22). The differences observed between etiologies could indicate that the sources of thrombus are potentially related to outcomes. Further large-sample trials should be conducted to investigate this further.

This study has some limitations. The study is limited by its retrospective pattern which has an inherent bias. The sample size of patients was limited due to the use of single-center data. Moreover, this study was performed in an Asian country, with ICAS being more prevalent compared with other counties in Europe and the United States. Therefore, there may be limited generalizability and the results should be examined in further studies. As the results showed no difference in functional outcomes between two etiologies, future research could focus on the etiology diagnosis approach or other influencing factors of outcomes.

## Conclusion

Etiology was not considered as an important factor in functional outcome, despite the differences in baseline characteristics and technical EVT approach. The current study of anterior circulation AIS-LVO patients supports that outcomes for those with ICAS are not significantly different from those with CE.

## Data availability statement

The original contributions presented in the study are included in the article/supplementary material, further inquiries can be directed to the corresponding author.

## Ethics statement

The studies involving human participants were reviewed and approved by the institutional ethics committee of Xuanwu Hospital, Capital Medical University. Written informed consent for participation was not required for this study in accordance with the national legislation and the institutional requirements.

## Author contributions

BY designed experiments. CM, WC, YH, YC, YW, JC, PG, FC, QM, and LJ organized clinical database. CM and WC contributed to the selection and data extraction processes of literature in meta-analysis. CM, WC, and YH wrote the manuscript. QT performed the statistical analysis. AD, RR, and WC revised the manuscript. All authors read and approved the submitted version.

## Conflict of interest

The authors declare that the research was conducted in the absence of any commercial or financial relationships that could be construed as a potential conflict of interest.

## Publisher’s note

All claims expressed in this article are solely those of the authors and do not necessarily represent those of their affiliated organizations, or those of the publisher, the editors and the reviewers. Any product that may be evaluated in this article, or claim that may be made by its manufacturer, is not guaranteed or endorsed by the publisher.
